# Supplementary material for: Study of real-time parameter measurement of ring rolling pieces based on machine vision
Source: PLoS One. 2024 Feb 23;19(2):e0298607. doi: 10.1371/journal.pone.0298607 (PMC10889852; doi:10.1371/journal.pone.0298607)
Supplement: Attachment — Submitted filename: Response to Reviewers.docx [file pone.0298607.s003.docx]

**Response to Reviewers**

Dear Editors and Reviewers:

Thank you for your letter and for the reviewer(s)’ comments concerning our manuscript entitled “Study of Real-time Parameter Measurement of Ring Rolling Pieces Based on Machine Vision” (Manuscript ID PONE-D-23-34868). Those comments are all valuable and helpful for revising and improving our paper. We have studied comments carefully and have made correction which we hope to meet with approval. Revised parts are marked in red in the paper. The main corrections in the paper and the responses to the reviewers and editor’s comments are as follows:

**Editor remarks:**

**1. Response to comment:** (Please ensure that your manuscript meets PLOS ONE's style requirements, including those for file naming.)

**Response:** Thank you very much. The revised manuscript can meet PLOS ONE's style requirements.

**2. Response to comment:** (Please note that PLOS ONE has specific guidelines on code sharing for submissions in which author-generated code underpins the findings in the manuscript. In these cases, all author-generated code must be made available without restrictions upon publication of the work.)

**Response:** Thank you very much. We have stored the code in the figshare public repository. DOI: 10.6084/m9.figshare.25052933.

**3. Response to comment:** (Note from Emily Chenette, Editor in Chief of PLOS ONE, and Iain Hrynaszkiewicz, Director of Open Research Solutions at PLOS: Did you know that depositing data in a repository is associated with up to a 25% citation advantage? If you’ve not already done so, consider depositing your raw data in a repository to ensure your work is read, appreciated and cited by the largest possible audience. You’ll also earn an Accessible Data icon on your published paper if you deposit your data in any participating repository.)

**Response:** Thank you. We have stored the raw data in the figshare public repository. DOI: 10.6084/m9.figshare.25052933.

**4. Response to comment:** (We note that the grant information you provided in the ‘Funding Information’ and ‘Financial Disclosure’ sections do not match.)

**Response:** Thank you. We have provided the correct grant numbers for the awards. This research was funded by the Jianghan University Science and Technology Innovation Project, China (No.2021kjzx011), and the Jianghan University Graduate Research Innovation Fund Project, China (No. KYCXJJ202306).

**5. Response to comment:** (Please confirm at this time whether or not your submission contains all raw data required to replicate the results of your study. Authors must share the “minimal data set” for their submission. PLOS defines the minimal data set to consist of the data required to replicate all study findings reported in the article, as well as related metadata and methods.)

**Response:** Thank you for your advice. We have stored the raw data in the figshare public repository. DOI: 10.6084/m9.figshare.25052933.

**6. Response to comment:** (PLOS requires an ORCID iD for the corresponding author in Editorial Manager on papers submitted after December 6th, 2016. Please ensure that you have an ORCID iD and that it is validated in Editorial Manager.)

**Response:** Thank you very much. We added their own ORCID ids after first author Xiaoge Fu and corresponding author Zhijiang Zuo.

**7. Response to comment:** (Please clarify the number of Figures uploaded in your manuscript and PDF file.)

**Response:** Sorry for our carelessness, we have carefully modified the number of charts in the file.

**8. Response to comment:** (Please include a copy of Table 6 which you refer to in your text on page 23.)

**Response:** Thank you for your careful reading. We have added the content of the table in the body.

**9. Response to comment:** (Please review your reference list to ensure that it is complete and correct.)

**Response:** Thank you. We conducted a review of the cited references, removed the retracted papers, and replaced them with relevant references.

**Reviewer 1:**

**1. Response to comment:** (The introduction section should be enhanced by incorporating insights from prior research, and it would benefit from the inclusion of recent citations pertaining to ring measurements.)

**Response:** Thank you for your advice. In the introduction, we add the research of Zheng Lu, Yu-cun Zhang, Bangguo Wang, Xianbin Fu, Yazid Saif and others on the measurement of ring parts, and add relevant citations.

**2. In the methodology section:**

**a. Response to comment:** (Can you provide more comprehensive details about the instruments and devices utilized for ring measurements in the methodology section?)

**Response:** Thank you very much. We have included a detailed description of the mechanical structure in "2.2 Mechanical structure design ", which explains the function of each module. In "4.1 Experimental platform construction", the hardware and equipment parameters required by the experiment platform are comprehensively introduced.

**b. Response to comment:** (How would including information about the measurement equipment's accuracy and calibration procedures contribute to strengthening the overall robustness of the study?)

**Response:** Thank you for your valuable advice. We have added the section "3.2 Camera calibration" to the text. Camera calibration is an important form of calibration procedure to ensure the accuracy and reliability of the measurement system. The process and result of camera calibration are also given.

**c. Response to comment:** (Could you offer clarification on the sample size and variability in the experiments to ensure a clearer understanding of the research parameters?)

**Response:** Thank you for your comments on our articles. According to your suggestion, we explained the reason for selecting the test ring in the measurement experiment, and carried out several measurements on the sample. The validity and stability of the experiment are verified by comparative analysis of the different conditions that affect the measurement results.

**d. Response to comment:** (In what ways can you elaborate on the data collection process, including the frequency of measurements and the specific conditions under which they were conducted?)

**Response:** Thank you for your advice. We have added detailed information about the vision equipment, including the measurement frequency and conditions, to the experimental platform construction.

**3. Response to comment:** (It is necessary to verify the formatting of the references and the spacing between sentences and references. Such as “measurement[16] line 60, 74 , and Lizhou[17] line 79”.)

**Response:** Thanks for your careful reading, we have verified the format of the references and cited them in the "PLoS" style using EndNote X9.

**4. Response to comment:** (Some typos need to change “information(Fig 5) line 206”.)

**Response:** Sorry, we have revised the article and corrected the part with mistakes.

**5. Response to comment:** (It is necessary to discuss how the algorithms and pre-processing steps selected affect the overall quality of the results. How do these steps help to improve measurement accuracy or expand the system's capabilities? This link should be highlighted explicitly.)

**Response:** Thank you very much for your advice. We added the result graph without image preprocessing in the "3.1 Image Preprocessing" section, and discussed the influence of no preprocessing steps on the measurement results. At the same time, literature [23] was added to further prove the importance of pre-treatment steps.

**6. Response to comment:** (Consider discussing the performance of various algorithms, if applicable. Is it possible to find algorithms that outperform others in specific scenarios? Exploring these comparative aspects can provide insights into the decision-making process for algorithm selection.)

**Response:** Thank you for your advice. We added the comparison of key algorithms in "3.1 Image Preprocessing", showed the comparison result graph, and expounded the basis of algorithm decision-making.

**7. Response to comment:** (Recommend potential future research directions, such as investigating the method's extension to address challenges associated with various types of metrology measurements or exploring how it interacts with modern manufacturing systems.)

**Response:** Thank you for your helpful advice. We have added a vision for the future at the end of the "5 Conclusions" section.

**8. Response to comment:** (The manuscript needs to be proofread to be more suitable for publishing.)

**Response:** Thank you for your advice. We have proofread the article and made changes to the relevant positions.

It is our pleasure to resubmit the revised manuscript to your journal. Thanks again.

Sincerely yours.

Wish you all the best!

Sincerely yours,

Xiaoge Fu, Han Li, Zhijiang Zuo, and Libo Pan
