# Supplementary material for: Study of real-time parameter measurement of ring rolling pieces based on machine vision
Source: PLoS One. 2024 Feb 23;19(2):e0298607. doi: 10.1371/journal.pone.0298607 (PMC10889852; doi:10.1371/journal.pone.0298607)
Supplement: Attachment — Submitted filename: Reviewer Comments to authors.docx [file pone.0298607.s002.docx]

Reviewer comments:

1. The introduction section should be enhanced by incorporating insights from prior research, and it would benefit from the inclusion of recent citations pertaining to ring measurements. You need to look for and add related this citation as DOI below” <https://doi.org/10.1016/j.measurement.2022.111072>, <https://doi.org/10.1007/s00170-022-10359-0> , <https://doi.org/10.1007/s11740-023-01232-4> , <https://doi.org/10.1371/journal.pone.0292814> ,<https://doi.org/10.3390/app11062601> , <https://doi.org/10.3390/s23177443>, “
2. In the methodology section:
a. Can you provide more comprehensive details about the instruments and devices utilized for circularity measurements in the methodology section?
b. How would including information about the measurement equipment's accuracy and calibration procedures contribute to strengthening the overall robustness of the study?
c. Could you offer clarification on the sample size and variability in the experiments to ensure a clearer understanding of the research parameters?
d. In what ways can you elaborate on the data collection process, including the frequency of measurements and the specific conditions under which they were conducted?
3. It is necessary to verify the formatting of the references and the spacing between sentences and references. Such as “measurement[16] line 60, 74 , and Lizhou[17] line 79”.
4. Some typos need to change “information(Fig 5) line 206”.
5. It is necessary to discuss how the algorithms and pre-processing steps selected affect the overall quality of the results. How do these steps help to improve measurement accuracy or expand the system's capabilities? This link should be highlighted explicitly.
6. Consider discussing the performance of various algorithms, if applicable. Is it possible to find algorithms that outperform others in specific scenarios? Exploring these comparative aspects can provide insights into the decision-making process for algorithm selection.
7. Recommend potential future research directions, such as investigating the method's extension to address challenges associated with various types of metrology measurements or exploring how it interacts with modern manufacturing systems.
8. The manuscript needs to be proofread to be more suitable for publishing.
